# Supplementary material for: Deep learning-based patient re-identification is able to exploit the biometric nature of medical chest X-ray data
Source: Sci Rep. 2022 Sep 1;12:14851. doi: 10.1038/s41598-022-19045-3 (PMC9434540; doi:10.1038/s41598-022-19045-3)
Supplement: Supplementary file 1 — Supplementary Table 1. [file 41598_2022_19045_MOESM1_ESM.pdf]

Deep Learning-based Patient Re-identification Is able to Exploit the Biometric Nature of Medical Chest X-ray Data

Kai Packhäuser[1,*], Sebastian Gündel[1], Nicolas Münster[1], Christopher Syben[1], Vincent Christlein[1], and Andreas Maier[1]

[1]Pattern Recognition Lab, Department of Computer Science, Friedrich-Alexander University Erlangen-Nürnberg, 91058 Erlangen, Germany
[*]kai.packhaeuser@fau.de

Supplementary Material

Supplementary Table 1. Additional patient verification results: Comparison of different data handling techniques (FTS and RNP), training set sizes N_s and learning rates η. We present the AUC (together with the lower and upper bounds of the 95 % confidence intervals from 10,000 bootstrap runs), the accuracy, the specificity, the recall, the precision, and the F1-score.

Data handling	N_s	η	AUC + 95 % CI	Accuracy ($\frac{TP+TN}{P+N}$)	Specificity ($\frac{TN}{N}$)	Recall ($\frac{TP}{P}$)	Precision ($\frac{TP}{TP+FP}$)	F1-score
FTS	100,000	10^{-4}	0.8509 $^{0.8532}_{0.8485}$	0.7461 ($\frac{74,605}{100,000}$)	0.8414 ($\frac{42,071}{50,000}$)	0.6507 ($\frac{32,534}{50,000}$)	0.8040 ($\frac{32,534}{40,463}$)	0.7193
		10^{-5}	0.7429 $^{0.7459}_{0.7399}$	0.6322 ($\frac{63,223}{100,000}$)	0.7924 ($\frac{39,619}{50,000}$)	0.4721 ($\frac{23,604}{50,000}$)	0.6945 ($\frac{23,604}{33,985}$)	0.5621
		10^{-6}	0.7257 $^{0.7289}_{0.7225}$	0.6702 ($\frac{67,020}{100,000}$)	0.6719 ($\frac{33,594}{50,000}$)	0.6685 ($\frac{33,426}{50,000}$)	0.6708 ($\frac{33,426}{49,832}$)	0.6696
		10^{-7}	0.7440 $^{0.7470}_{0.7409}$	0.6741 ($\frac{67,410}{100,000}$)	0.7002 ($\frac{35,008}{50,000}$)	0.6480 ($\frac{32,402}{50,000}$)	0.6837 ($\frac{32,402}{47,394}$)	0.6654
	200,000	10^{-4}	0.8886 $^{0.8906}_{0.8866}$	0.7920 ($\frac{79,203}{100,000}$)	0.8547 ($\frac{42,736}{50,000}$)	0.7293 ($\frac{36,467}{50,000}$)	0.8339 ($\frac{36,467}{43,731}$)	0.7781
		10^{-5}	0.8158 $^{0.8185}_{0.8132}$	0.6758 ($\frac{67,582}{100,000}$)	0.8574 ($\frac{42,869}{50,000}$)	0.4943 ($\frac{24,713}{50,000}$)	0.7761 ($\frac{24,713}{31,844}$)	0.6039
		10^{-6}	0.7615 $^{0.7645}_{0.7586}$	0.6755 ($\frac{67,552}{100,000}$)	0.7448 ($\frac{37,242}{50,000}$)	0.6062 ($\frac{30,310}{50,000}$)	0.7038 ($\frac{30,310}{43,068}$)	0.6514
		10^{-7}	0.7526 $^{0.7556}_{0.7495}$	0.6819 ($\frac{68,194}{100,000}$)	0.6791 ($\frac{33,957}{50,000}$)	0.6847 ($\frac{34,237}{50,000}$)	0.6809 ($\frac{34,237}{50,280}$)	0.6828
	400,000	10^{-3}	0.9541 $^{0.9552}_{0.9529}$	0.8852 ($\frac{88,519}{100,000}$)	0.8655 ($\frac{43,275}{50,000}$)	0.9049 ($\frac{45,244}{50,000}$)	0.8706 ($\frac{45,244}{51,969}$)	0.8874
		10^{-5}	0.8518 $^{0.8542}_{0.8494}$	0.7450 ($\frac{74,498}{100,000}$)	0.8410 ($\frac{42,050}{50,000}$)	0.6490 ($\frac{32,448}{50,000}$)	0.8032 ($\frac{32,448}{40,398}$)	0.7179
		10^{-6}	0.7417 $^{0.7449}_{0.7386}$	0.6572 ($\frac{65,715}{100,000}$)	0.7152 ($\frac{35,761}{50,000}$)	0.5991 ($\frac{29,954}{50,000}$)	0.6778 ($\frac{29,954}{44,193}$)	0.6360
		10^{-7}	0.7306 $^{0.7337}_{0.7275}$	0.6567 ($\frac{65,670}{100,000}$)	0.6801 ($\frac{34,006}{50,000}$)	0.6333 ($\frac{31,664}{50,000}$)	0.6644 ($\frac{31,664}{47,658}$)	0.6485
	800,000	10^{-3}	0.9826 $^{0.9833}_{0.9820}$	0.9324 ($\frac{93,238}{100,000}$)	0.9393 ($\frac{46,966}{50,000}$)	0.9254 ($\frac{46,272}{50,000}$)	0.9385 ($\frac{46,272}{49,306}$)	0.9319
RNP		10^{-4}	**0.9940** $^{0.9944}_{0.9937}$	0.9555 ($\frac{95,545}{100,000}$)	0.9822 ($\frac{49,111}{50,000}$)	0.9287 ($\frac{46,434}{50,000}$)	0.9812 ($\frac{46,434}{47,323}$)	0.9542
		10^{-5}	0.9278 $^{0.9294}_{0.9262}$	0.8339 ($\frac{83,392}{100,000}$)	0.8946 ($\frac{44,732}{50,000}$)	0.7732 ($\frac{38,660}{50,000}$)	0.8801 ($\frac{38,660}{43,928}$)	0.8232
FTS		10^{-5}	0.9200 $^{0.9217}_{0.9182}$	0.8215 ($\frac{82,153}{100,000}$)	0.8888 ($\frac{44,440}{50,000}$)	0.7543 ($\frac{37,713}{50,000}$)	0.8715 ($\frac{37,713}{43,273}$)	0.8087
		10^{-6}	0.8669 $^{0.8692}_{0.8646}$	0.7752 ($\frac{77,519}{100,000}$)	0.8165 ($\frac{40,823}{50,000}$)	0.7339 ($\frac{36,696}{50,000}$)	0.7999 ($\frac{36,696}{45,873}$)	0.7655
		10^{-7}	0.8126 $^{0.8152}_{0.8099}$	0.7247 ($\frac{72,468}{100,000}$)	0.7502 ($\frac{37,509}{50,000}$)	0.6992 ($\frac{34,959}{50,000}$)	0.7368 ($\frac{34,959}{47,450}$)	0.7175
